# Incomplete vascular ring of the aortic arch presenting with dysphagia in an adult: case report

**DOI:** 10.11604/pamj.2023.45.183.38569

**Published:** 2023-08-25

**Authors:** Ndubueze Ezemba, Augustine Chukwudi Onuh, Uchenna Simon Onoh

**Affiliations:** 1 Division of Cardiothoracic Surgery, National Cardiothoracic Centre of Excellence, University of Nigeria Teaching Hospital, Ituku/Ozalla, Enugu, Nigeria,; 2 Department of Radiation Medicine, University of Nigeria Teaching Hospital, Ituku/Ozalla, Enugu, Nigeria

**Keywords:** Vascular ring, dysphagia lusoria, aberrant right subclavian artery, aortic arch anomaly, case report

## Abstract

Anomalies of the arterial branches of the arch of the aorta are rare, with the aberrant right subclavian artery being the most common of this anomaly. Majority of the anomalies are asymptomatic and often discovered as incidental findings. In the great majority of the symptomatic cases, the presentation may be either with breathlessness or dysphagia or both. This is in addition to the nature of the intrinsic arterial disease of the aberrant vessel, especially in adult patients; and unless borne in mind, the diagnosis is often missed leading to delays and wrong treatment. In this report we present a case of dysphagia in an adult male Nigerian initially diagnosed as œsophageal stricture from herbal potion ingestion but review of his imaging investigations gave a final diagnosis of dysphagia lusoria from an aberrant right subclavian artery. The difficulty in making a diagnosis and the need for a multidisciplinary review of the imaging investigations are highlighted. The patient was successfully treated by a combined trans-thoracic and cervical approach with division and re-implantation of the aberrant vessel unto the right common carotid artery. He has remained symptom-free for 2 years after surgery. Although the great majority of these anomalies are often asymptomatic, it is important they are borne in mind both in imaging investigations as well as in procedures involving structures in the upper visceral mediastinum. Various surgical approaches have been documented in the management of symptomatic ones; it is however recommended that options that ensure revascularization of the affected limb be selected.

## Introduction

Anomalies of the arch of the aorta are rare, with the majority manifesting as vascular rings which may be complete or incomplete [1]. One example of an incomplete vascular ring is the aberrant right subclavian artery (ARSA) arising from the left-sided aortic arch. First described by David Bayford in 1761, of a fatal case of ‘obstructed deglutition’, in a 62-year old woman who had life-long difficulty in swallowing, he also coined the term ‘dysphagia lusoria’ [2]. The prevalence of ARSA is estimated at about 0.5% and it is the most common anomaly of the aortic arch [3]. The anomaly which may be isolated or syndromic is thought to be slightly more common in females; and not infrequently arises from an aortic arch diverticulum called Kommerell diverticulum [3]. According to the typing system of Adachi and Williams, based on the anatomic morphology of the arch, ARSA is a type G aortic arch of which there are four variants: G, CG, H, and N. Type G represents a left-sided arch having the ARSA as the 4^th^ branch after the left subclavian artery (LSCA) while type N is a mirror image of the former in a right-sided arch. In type CG, the ARSA arises in a left-sided arch as the 5^th^ branch after the LSCA and with the left vertebral artery arising direct from the arch before the LSCA; and in type H it is the last branch after the LSCA in an arch with bicarotid trunk. Kieffer E, *et al*. [4] proposed a clinical grouping, with a bearing on treatment options, based on the characteristics of the aberrant artery. In the clinical grouping, Groups I and II represent nonaneurysmal ARSA with normal, and stenotic artery respectively; while Groups III and IV denote aneurysmal ARSA without or with involvement of the thoracic aorta respectively. The majority of the anomalies are asymptomatic and often discovered as incidental findings. The course of the aberrant vessel in relation to the nearby mediastinal structures determines the mode of presentation. In the great majority of the symptomatic cases, the presentation may be with breathlessness or dysphagia or both [5]. Presentation in adulthood is often associated with wrong/delayed diagnosis and inappropriate treatment [3,5]. In this report we present a case of dysphagia in an adult male Nigerian initially diagnosed as œsophageal stricture from herbal potion ingestion but review of his imaging investigations gave a final diagnosis of dysphagia lusoria due to an incomplete vascular ring as a result of an aberrant right subclavian artery arising from a left-sided aortic arch. The tell-tale findings that should raise a suspicion of such a lesion are highlighted as well as the various treatment options.

## Patient and observation

**Patient information**: a 28-year old man, member of the Jehovah´s Witness religion, presented to us on 31^st^ August, 2020 on referral from the Otolaryngology Department of the hospital on account of a 2-month history of grade 2 progressive dysphagia, with the feeling of foreign body sensation in the throat unassociated with odynophagia, haematemesis, regurgitation nor hoarseness but with occasional relief following drinking of large volumes of water. There was an associated history of 4Kg weight loss and occasional burning pain at the mid back. There was no history of ingestion of corrosives though he admitted to occasional ingestion of herbal potions; and although he usually took about 12 units of alcohol/week with occasional spirit, he did not smoke. He had been treated as a case of gastro-œsophageal reflux disease with no success.

**Clinical findings**: clinical examination was unimpressive apart from his weighing 58.5Kg as against his usual 62Kg. The initial working diagnosis was that of œsophageal stricture possibly from herbal potion ingestion.

**Figure 1:**
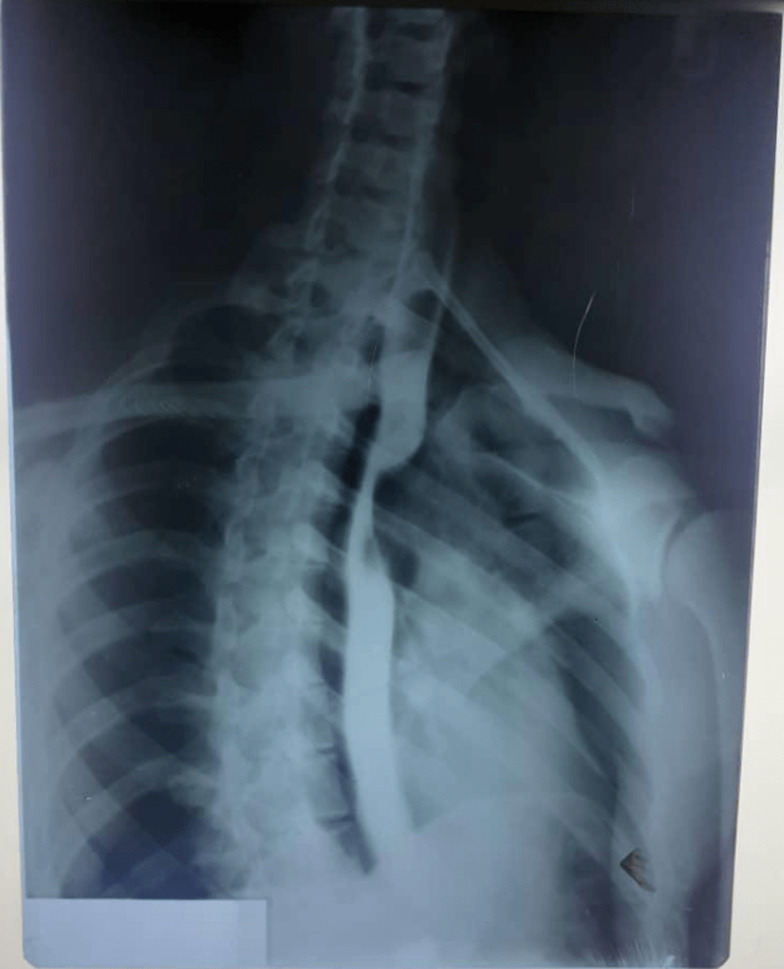
barium œsophagogram, note the severe œsophageal stenosis at the level of the aortic arch, thoracic vertebra 4/5, caused by an extrinsic mass

**Figure 2:**
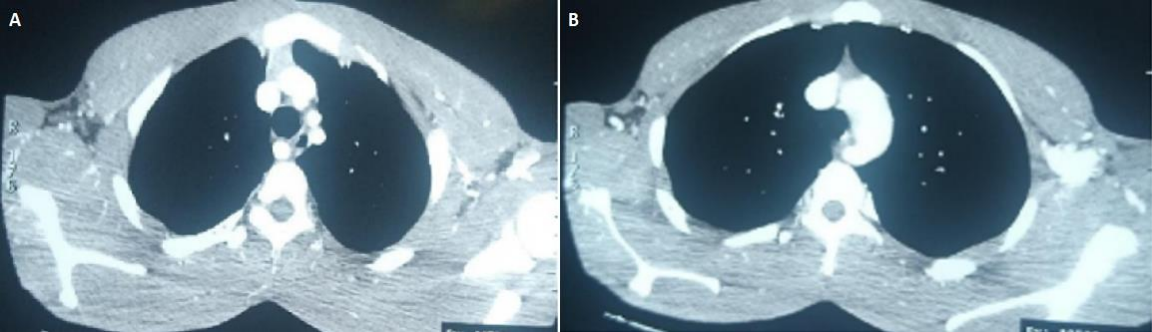
A) four arch vessels are noted; the 4^th^ is seen in the posterior mediastinum just in front of the 4^th^ thoracic vertebrum; B) at the level of the aortic arch, the 4^th^ vessel is seen arising from the arch and coursing behind the œsophagus and anterior to the spine

**Diagnostic approach**: the contrast œsophagogram showed extrinsic compression of the upper thoracic œsophagus about the level of the arch of the aorta, Figure 1. This was further investigated by a computerized tomographic scan of the chest (CT-Chest) done a week later and which was initially passed as normal. However, the finding of a contrast-enhancing lesion of unclear nature anterior to the 4^th^ thoracic vertebrum prompted a review of the scan with the radiologists during which the earlier described contrast-enhancing lesion was discovered to be an ARSA, Figure 2 (A,B).

**Presumptive**: œsophageal stricture secondary to herbal potion ingestion.

**Final**: aberrant right subclavian artery arising as a fourth branch from the aortic arch and running retro-œsophageal to the œsophagus.

**Therapeutic intervention**: the patient was subsequently operated upon on the 8^th^ October, 2020 i.e. 5 weeks later in a surgery involving an œsophagoscopy, left thoracotomy and right supraclavicular dissection.

***œsophagoscopy***: at œsophagoscopy, extrinsic pulsation was noted at the left side of the œsophagus at 25cm mark from the upper incisor and with deviation of the œsophagus to the right. The œsophageal mucosa was normal and the scope was readily advanced to the gastro-œsophageal junction with ease.

**Figure 3:**
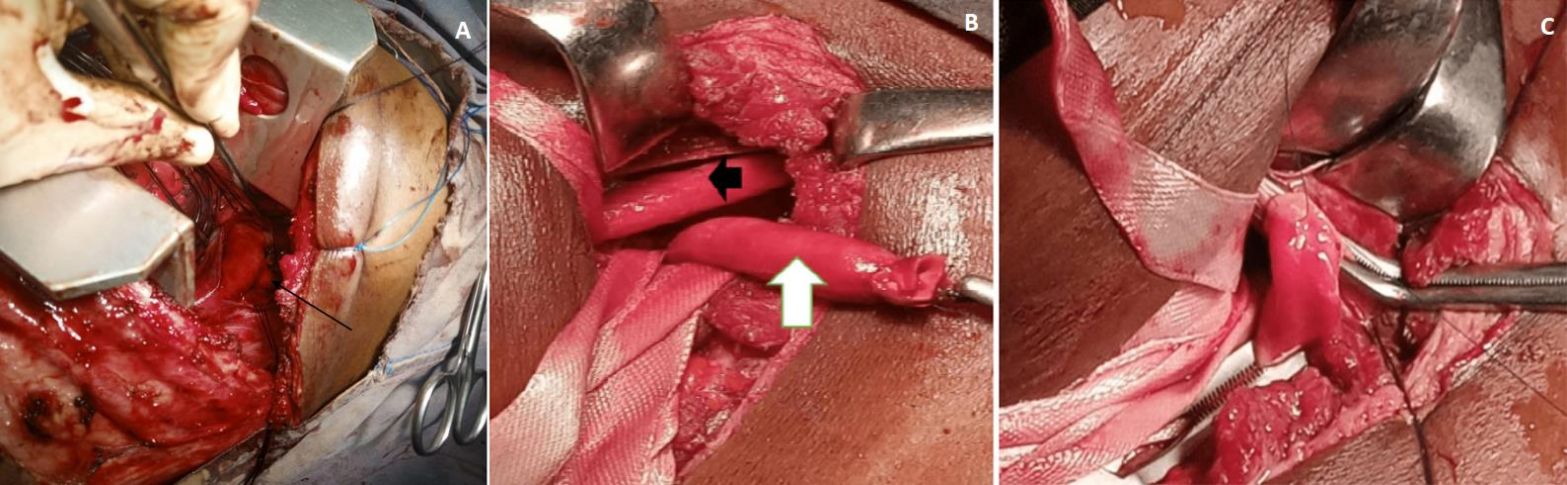
A) intra-operative (left posterolateral thoracotomy), the forceps points to the left subclavian artery; the arrow points to the aberrant right subclavian artery; B) intra-operative, right supraclavicular incision (white arrow = mobilized aberrant right subclavian artery), (black arrow = right common carotid artery); C) intra-operative: aberrant right subclavian artery being anastomosed to the right common carotid artery

***Thoracotomy***: following œsophagoscopy, he was placed on a right lateral position and via a left posterolateral thoracotomy, the pleural cavity was entered through the 4^th^ intercostal space. The lung was retracted inferiorly and anteriorly and the arch of the aorta exposed. The mediastinal pleura overlying the proximal portion of the descending aorta and the arch of the aorta was incised open thereby exposing the left common carotid artery (LCCA), the left subclavian artery (LSCA) and the aberrant right subclavian artery (ARSA), (Figure 3A). Test clamping of the latter with disappearance of the right finger oxygen pulse oximetry reading, and absence of blood pressure reading on the right upper limb confirmed it to be the aberrant vessel. This was seen to run from the left side to behind the œsophagus. It was mobilised to well behind the œsophagus and encircled, (Figure 3B). It was then clamped and divided with the proximal end repaired flush to the aorta using running 5/0 polypropylene suture and the distal stump ligated and transfixed. The mediastinal pleura was re-approximated, haemostasis secured and the lung re-expanded. The thoracotomy wound was closed in the usual manner with a size 28-gauge thoracic drain and subcuticular skin closure.

***Neck incision***: following re-positioning to supine, the neck was hyperextended and rotated to the left. After prepping and draping, a 5-cm transverse right supraclavicular incision was made; dividing the platysma, clavicular head of the sternocleidomastoid muscle (SCM) and partially incising the sternal head of the same SCM. The carotid sheath was incised open, the right internal jugular vein (IJV), CCA and vagus nerve identified and safe-guarded. The ARSA was mobilised from within the chink between the right CCA and IJV. After heparinization with 5000iu of unfractionated intravenous heparin, an end-to-side right subclavian artery to CCA anastomosis was effected using running 5/0 polypropylene suture leading to a restoration of oxygen pulse oximetry and blood pressure reading to the right upper limb, (Figure 3C). The cervical wound was closed in the usual manner with subcuticular skin suture. The total estimated blood loss was 350mls and there was no intra-operative blood transfusion.

***Post-operative care:*** following reversal of anaesthesia and extubation, he spent 14 hours in the intensive care unit. The rest of the post-operative ward care was uneventful. He was discharged on the 6^th^ post-operative day.

**Figure 4:**
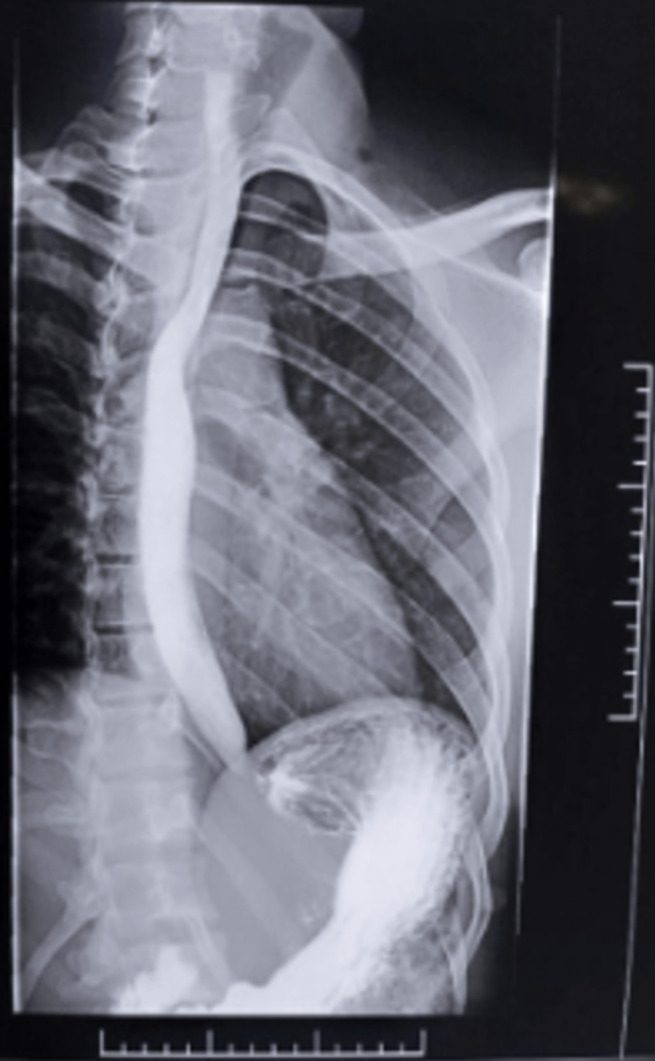
barium œsophagogram 2 weeks post operation; œsophageal stenosis resolved

**Follow up and outcome of intervention**: a repeat barium œsophagogram 2-weeks post-surgery showed normal œsophagus, Figure 4. At 3-months post-surgery, he had returned to his pre-symptom weight of 62.0Kg and at 12 months after, he had remained well, swallowing freely and weighed 63.0Kg. Twenty-four months after surgery he remained symptom free.

**Patient perspective**: the University of Nigeria Teaching Hospital (UNTH) carefully handled my case even though it took time to ascertain what my problem was. I later understood that such passage of time was to find out the real course of my problem. I also received an extensive care at UNTH even as they were willing to operate on me without blood transfusion.

**Informed consent:** the patient signed and gave consent for the information used in this case. The consent is available on request.

## Discussion

Although the great majority of ARSA are asymptomatic, often picked up as incidental findings, the symptomatic ones tend to present at about the middle age [3]. The course of the aberrant vessel in relation to the contiguous mediastinal structures generally determines the clinical symptoms. Where the vessel runs anterior to the trachea, the symptom is usually that of breathlessness as a result of airway compression. This is particularly so in infants and children in whom the tracheal cartilage, not firm enough, allows for easy compression of the airway. If, as in our case, the vessel runs posterior to the œsophagus and which is the usual course in most cases, symptomatic ones will frequently manifest as dysphagia and in some with recurrent foreign body impaction. A combination of breathlessness and dysphagia will occur when the ARSA runs in between the trachea and œsophagus. Further clinical manifestation will be dependent on the clinical grouping of the ARSA as proposed by Keiffer, *et al*. [4], with ischaemic signs in stenotic vessels; and embolic phenomena in aneurysmal variants. Our case, being Adachi and Williams type G and Keiffer group I had no additional symptoms besides the dysphagia.

Because ARSA is rare and often not thought about in the differentials of breathlessness and/or dysphagia, it is not surprising that the tell-tale signs in imaging investigations that should raise a suspicion of it are often overlooked. This was the case with our patient where the Barium œsphagogram and the mediastinal window of the chest CT were initially passed as normal. Whereas the aortic arch normally makes an indentation on the œsophagus at the level of the 4^th^ thoracic vertebrum, such indentation should not lead to prestenotic dilatation. Where attention is paid to the arch of the aorta, any variation from the usual 3 vessels arising therefrom should be further investigated. A 4^th^ vessel therefrom when traced may be observed to run either anterior to or behind the mediastinal structures; and in some cases in between. Endoscopy will usually show left-sided extrinsic pulsation of the œsophagus at about the level of the aortic arch. The presence of this anomaly should be borne in mind in procedures such as tracheostomy, right transradial artery coronary angiography, and surgeries involving the œsophagus whether congenital or acquired. Torrential bleeding in cases of tracheostomy, especially by percutaneous routes, massive upper gastro-intestinal bleeding, aortic dissection following transradial artery coronary angiography as well as difficulties with certain operations on the œsophagus have all been reported as a complication of undiagnosed ARSA [6-8].

The treatment of symptomatic ARSA has evolved over time. In general, patients with mild symptoms and those unwilling to undergo surgery are managed medically with diet modification. When symptoms are significant (intractable breathlessness, weight loss from dysphagia, limb ischaemia, embolic phenomena, associated aortic aneurysm and Kommerell´s diverticulum greater than 1.5cm in diameter or enlarging) such patients should be offered surgery if there are no contraindications. Surgical options have evolved over time; from simple division and ligation as popularized by Gross to endoscopic and hybrid procedures [9,10]. Simple division and ligation, although acceptable in young children, may not be advisable in symptomatic adults. This is especially true in those with Keiffer groups II -IV. Even in type G-I, as in our case, it is advisable to re-establish blood supply to the ipsilateral limb by anastomosing to the common carotid artery. Moreover, in simple division, it is important that the proximal stump be ligated flush to the aorta so as to avoid the development of stump aneurysm with recurrence of symptoms. This is one argument against some surgical routes such as the right-sided thoracotomy. Aneurysmal ARSAs are better managed by open surgery via a left thoracotomy and sometimes with a left sided heart bypass; reserving endovascular approach for the very frail and high risk patients.

## Conclusion

Anomalies of the branching pattern of the aortic arch are rare. Although the great majority of these anomalies are often asymptomatic, it is important they are borne in mind both in imaging investigations as well as in procedures involving structures in the upper visceral mediastinum. Various surgical approaches have been documented in the management of symptomatic ones; it is however recommended that options that ensure revascularization of the affected limb be selected.
